# Melatonin: a promising neuroprotective agent for cerebral ischemia-reperfusion injury

**DOI:** 10.3389/fnagi.2023.1227513

**Published:** 2023-08-04

**Authors:** Majid Tozihi, Behrouz Shademan, Hadi Yousefi, Cigir Biray Avci, Alireza Nourazarian, Gholamreza Dehghan

**Affiliations:** ^1^Department of Biology, Faculty of Natural Sciences, University of Tabriz, Tabriz, Iran; ^2^Stem Cell Research Center, Tabriz University of Medical Sciences, Tabriz, Iran; ^3^Department of Basic Medical Sciences, Khoy University of Medical Sciences, Khoy, Iran; ^4^Department of Medical Biology, Faculty of Medicine, EGE University, Izmir, Türkiye

**Keywords:** melatonin, cerebral ischemia-reperfusion injury, reactive oxygen species (ROS), ischemic strokes, neuroprotective agent

## Abstract

Cerebral ischemia-reperfusion (CIR) injury is initiated by the generation of reactive oxygen species (ROS), which leads to the oxidation of cellular proteins, DNA, and lipids as an initial event. The reperfusion process impairs critical cascades that support cell survival, including mitochondrial biogenesis and antioxidant enzyme activity. Failure to activate prosurvival signals may result in increased neuronal cell death and exacerbation of CIR damage. Melatonin, a hormone produced naturally in the body, has high concentrations in both the cerebrospinal fluid and the brain. However, melatonin production declines significantly with age, which may contribute to the development of age-related neurological disorders due to reduced levels. By activating various signaling pathways, melatonin can affect multiple aspects of human health due to its diverse range of activities. Therefore, understanding the underlying intracellular and molecular mechanisms is crucial before investigating the neuroprotective effects of melatonin in cerebral ischemia-reperfusion injury.

## 1. Introduction

Stroke is the leading cause of death in adults, with approximately 85% of strokes being ischemic in origin, and is associated with functional impairment and disability. Ischemic stroke occurs when a thrombus blocks the arteries supplying blood to the brain (Shademan et al., 2021) and is the most common type of stroke. The ischemic phase is followed by cerebral reperfusion, a more severe form of brain injury resulting from the restoration of blood flow. Several factors contribute to the detrimental effects of reperfusion, including oxidative stress, disruption of the blood-brain barrier, accumulation of platelets, complement cells, and leukocytes in the circulation, their invasion of the brain parenchyma, and hemorrhagic transformation (Lin et al., 2016; Shademan et al., 2021). In addition, ischemia leads to the development of secondary damage caused by excitotoxicity, excessive calcium levels in neurons, apoptosis, autophagy, and neuroinflammation. The nuclear transcription factor kappa B (NF-κB) controls the primary inflammatory response and initially exists in the cytoplasm complexed with protein inhibitor alpha (IαIp). During ischemia, NF-κβ is produced and translocated to the nucleus where it stimulates the production of pro-inflammatory cytokines (Liu et al., 2023a). Ischemia/reperfusion has been shown to induce apoptosis, a form of cell death characterized by significantly less necrosis Cytotoxic factors such as oxidative stress can also induce apoptosis (Kalogeris et al., 2016; Ajoolabady et al., 2022).

The preferred treatment approaches for ischemic stroke include rapid vascular recanalization and the use of recombinant tissue plasminogen activator (tPA) whenever possible (Liu L. et al., 2019). However, not all patients can benefit from these therapies due to the limited time window for effective treatment. In addition, restoring blood flow to the brain can paradoxically lead to reperfusion injury, resulting in the accumulation of reactive oxygen species (ROS) in brain cells and irreparable damage to brain tissue (Caplan et al., 2023).

Most mammals, including humans, produce melatonin, which functions as an amine hormone and scavenger of free radicals. This synthesis occurs primarily in the pineal gland (Ebrahimi et al., 2023; Suzen, 2023). Previous studies have shown that melatonin has several pharmacological effects, including reducing oxidative stress levels, regulating circadian rhythms, preventing apoptosis, and reducing inflammation (Liu et al., 2023b; Rahman and Yang, 2023). The neuroprotective benefits of melatonin in experimental stroke models are well documented. Several studies have demonstrated a reduction in infarct size and cerebral edema volume, along with improved neurological function (Wang et al., 2022; Pluta et al., 2023), indicating the presence of these beneficial effects. Notably, patients with ischemic stroke tend to have lower levels of melatonin. The average nocturnal melatonin level in healthy individuals was found to be 69. 70 pg/ml in the systemic circulation. However, patients with stroke had lower melatonin levels, with an average of 48. 1 ± 35. 9 pg/ml. The reduction in melatonin levels was found to be associated with the occurrence of stroke. In addition, a cross-sectional case-control study found an association between a 1 pg/mL decrease in melatonin levels and a greater than 2% increase in stroke risk (Atanassova et al., 2009). Melatonin has been shown in recent cases to be effective in preventing ischemia-reperfusion (I/R) injury, especially cerebral I/R injury (Liu L. et al., 2019; Mao et al., 2020). A growing body of research suggests that melatonin reduces I/R damage by decreasing cellular oxidative stress, reducing cellular calcium excess, preventing endoplasmic stress, and preventing mitochondrial apoptosis (Yu et al., 2016; Abolhasanpour et al., 2021). Its many functions may have an impact on human health. However, the underlying biochemical and molecular processes by which melatonin exerts its neuroprotective effects in cerebral I/R injury remain to be explored.

## 2. Pathophysiology of CIR injury (IRI)

During reperfusion, oxygen and substrates necessary for tissue healing are delivered to the ischemic tissue, while harmful metabolites are removed. However, reperfusion can lead to dangerous events that exacerbate tissue damage. Ischemia-reperfusion injury is characterized by a decline in tissue function after reperfusion. The complex pathophysiology of ischemia-reperfusion injury includes increased levels of reactive oxygen species (ROS), neutrophil activation, complement activation, the involvement of cytokines and other inflammatory mediators, and the presence of vasoactive molecules such as nitric oxide (NO) and endothelin. Ischemia-reperfusion injury can result in a variety of outcomes, including reversible cellular dysfunction, local and distant tissue damage, multiple organ failure, and even death.

### 2.1. Microvascular reperfusion defects

The neurovascular unit (NVU) consists of brain capillaries, pericytes, astrocytes, extracellular matrix (ECM), microglia, and neurons (Iadecola, 2017). The specialized endothelium in these capillaries forms the blood-brain barrier (BBB), a tightly regulated interface between the brain microvasculature and CNS tissue. The BBB selectively allows the passage of water and nutrients while preventing the entry of toxins and pathogens through a network of tight junctions and specialized transporters (Cummins, 2011; Knowland et al., 2014). Endothelial cell activation, which occurs in the lining of blood vessels, plays a critical role in the development of microvascular injury. Factors such as adhesion molecules and chemokines are upregulated on endothelial cells, facilitating the adherence and migration of leukocytes into brain tissue. This immune cell recruitment contributes to the inflammatory response and tissue damage. Ischemia-reperfusion (I/R) injury leads to decreased endothelial barrier function, increased permeability, and cellular edema, resulting in microthrombi, microvascular occlusion, and reduced blood supply, exacerbating reperfusion injury (Gao et al., 2019; Rios-Navarro et al., 2019). Apoptosis has been observed within 5 min of reperfusion in studies using isolated rat tissue (Deussen, 2018). No-reflow, a phenomenon in which blood flow is not restored despite vessel reopening, involves several mechanisms including capillary blockage by leukocytes or clumped red blood cells, capillary compression by swollen endothelial cells and cardiomyocytes, and impaired vasomotion (Li et al., 2022). However, treatments that target these factors to reduce injury, including vasodilators, antithrombotics, and anti-inflammatory agents, have shown limited success (Nazir et al., 2016; McCartney et al., 2019).

Overall, microvascular pathology in cerebral ischemia-reperfusion injury involves vascular disruption, increased blood-brain barrier permeability, oxidative stress, inflammation, and immune cell activation. Understanding these mechanisms is critical for the development of therapeutic strategies to mitigate the damage caused by I/R injury. Further research is needed to fully understand the mechanisms underlying microvascular dysfunction after I/R injury.

### 2.2. Excessive inflammatory response

After cerebral ischemia, microglia are activated and migrate to the ischemic area to remove toxins and maintain tissue homeostasis. In addition, microglia and recruited macrophages participate in the removal of cellular debris and dying cells through a process called phagocytosis (Kuznetsov et al., 2019). However, when microglia become hyperactive, they secrete pro-inflammatory cytokines such as TNF-α, IL-1, and IL-6, leading to uncontrolled inflammation, exacerbation of tissue loss, and induction of neurogenesis and cell death (Liu N. B. et al., 2019). Therefore, it is crucial to limit microglial hyperactivation and inflammation in the early stages of acute ischemic stroke to significantly reduce brain damage (Birnbaum et al., 2019).

After stroke, microglial activation results in the release of several inflammatory mediators, many of which have cytotoxic or cytoprotective properties. Activated microglia (M1) often release inflammatory and neurotoxic compounds such as reactive oxygen species (ROS), nitric oxide, TNF-α, and IL-6. These molecules and their derivatives, such as peroxynitrite, have the potential to damage neurons. Conversely, alternatively activated microglia (M2) promote tissue healing by secreting growth factors and anti-inflammatory molecules (Liu N. B. et al., 2019). Recent research has demonstrated the significant impact of microglial polarization on the recovery of brain function after cerebral ischemia. This growing body of evidence highlights how microglial cell behavior can influence neuronal death and neurogenesis (Hu et al., 2015; Jolivel et al., 2015).

Microglial polarization is characterized by the production of different cytokines and the activation of different transcription factors. For example, M1 polarization is associated with the stimulation of pro-inflammatory cytokines such as TNF-α, IL-1, IL-6, and IL-12 and the activation of the transcription factors STAT1 and NF-κB (Amanakis et al., 2019; Patel and Strong, 2019). Conversely, M2 polarization is known to produce cytokines such as TGF-β, CCL18, and IL-1Ra, which contribute to the reduction of inflammation. Activation of the transcription factor STAT6 can also increase expression of the mannose receptor CD206 and other markers of M2 polarization (Neher et al., 2013).

Targeting specific pathways and transcription factors may prove beneficial in promoting the desired M2 polarization state.

### 2.3. Cellular oxidative stress

Oxidative stress is primarily induced by the presence of free radicals and reactive oxygen species (ROS). It is recognized as a significant contributor to the deterioration and impairment of cellular processes within an organism (Lucas et al., 2006). Mitochondrial dysfunction and oxidative stress are two key factors contributing to the exacerbation of long-term damage after cerebral ischemia and reperfusion. Disturbances in energy metabolism disrupt the balance between oxidation and antioxidation in the body, resulting in the overproduction of ROS and hydroxyl radicals in neuronal mitochondria. These molecules can further damage the brain, causing oxidative stress and neuronal death. Mitochondrial dysfunction and oxidative stress have a significant impact on the brain and can lead to long-term damage after stroke. Therefore, it is critical to identify approaches to mitigate the effects of oxidative stress and restore normal mitochondrial function to reduce brain damage and facilitate recovery (Boutin et al., 2001).

Brain ischemia/reperfusion disrupts oxidative phosphorylation, leading to an increase in reactive oxygen species (ROS) and lipid peroxidation (Patel et al., 2013). ROS primarily damage cells through two mechanisms: (1) by causing lipid peroxidation in mitochondrial membranes through a reaction with polyunsaturated fatty acids (Shin et al., 2014), and (2) by cross-linking macromolecules such as DNA, RNA, polysaccharides, and amino acids, resulting in the loss of their original activity or function (Yao et al., 2023). (3) Endothelial cell damage: Free radicals have a high reactivity toward oxidized lipids and proteins, which can lead to endothelial cell damage. This damage leads to increased permeability of the blood-brain barrier (Orekhov et al., 2019); (4) Mediating Inflammation and Immune Response: Free radicals stimulate the expression of cytokines and adhesion molecules, thereby promoting inflammation and immune responses. This process can exacerbate reperfusion injury to brain tissue (Zhang et al., 2020); (5) Promotion of polymerization and degradation of polysaccharide molecules: Free radicals have the ability to affect polysaccharides by promoting their polymerization or degradation. This can affect several cellular functions (Sharma and Mehdi, 2023); and (6) Increase the release of excitatory amino acids (EAA), which promote the appearance of delayed neuronal death after cerebral ischemia (Wu et al., 2018).

To protect cell structure and function, an endogenous antioxidant system maintains a dynamic balance between free radical generation and scavenging. The total antioxidant capacity (TAC) of the human body is determined by considering antioxidant activity, free radical metabolism, and antioxidant systems (Angelova et al., 2023). Higher serum TAC levels have been associated with mortality in patients with severe ischemic stroke, making it a potential predictive biomarker (Mukherjee et al., 2019). TAC includes both enzymatic and non-enzymatic systems. Enzymatic systems include glutathione peroxidase (GSHPx), catalase, glutathione S-transferase (GST), paraoxonase (PON), superoxide dismutase (SOD), thioredoxin (Trx), and others. Non-enzymatic systems include carotenoids, vitamins A, C, and E, and glutathione (GSH) (Li et al., 2019a). Modulation of TAC has been shown to prevent neuronal damage in cerebral ischemia-reperfusion injury (CIRI) (Li et al., 2019a; Wang Y. et al., 2019). For example, Lin et al. demonstrated a significant reduction in neuronal damage in the CA1 area of the hippocampus by administering Lin as a TAC modulator after cerebral ischemia (Xu et al., 2018).

### 2.4. Autophagy

Autophagy serves essential functions in replacing damaged organelles, mobilizing amino acids during nutrient deprivation, and facilitating tissue regeneration during growth or in response to hormonal cycles (Lu et al., 2010). While autophagy plays a critical role in maintaining cellular homeostasis, excessive or dysregulated autophagic responses can have negative consequences. Excessive autophagy can deplete cellular resources, act as a sink, and potentially compromise cellular functions (Zhang et al., 2015; Kruk et al., 2022) In addition, in certain cases, autophagy can contribute to the accumulation of pathogenic amyloid-beta (Aβ) proteins associated with neurodegenerative diseases such as Alzheimer’s disease (Lorente et al., 2016). Autophagic stress involves the dynamic interplay of cell formation, maturation, and degradation and helps to explain the seemingly contradictory roles that autophagy-related processes play in homeostasis, adaptation, degeneration, and even cell death. Recent *in vitro* and *in vivo* studies have provided increasing evidence that autophagy plays a critical role in the regulation and control of inflammatory mediators during inflammatory processes (Deng et al., 2015; Kryl’skii et al., 2019).

The role of autophagy in ischemia/reperfusion (I/R) injury remains controversial, with some investigators suggesting its protective effects and others claiming its destructive effects (Lin et al., 2015; Zha et al., 2021; Magen et al., 2022). Autophagy plays a critical role in neuronal function and disease pathogenesis (Antonucci et al., 2015), but current research in this area requires further investigation (Moradi Majd et al., 2020). Shi et al. demonstrated that autophagy activity was protective against ischemic hypoxic injury (Qian et al., 2017). However, Zhou and colleagues proposed that autophagy may support the apoptotic process (Bussi et al., 2017). To understand these conflicting findings, it is important to analyze the level of autophagy itself, as excessively high or low levels may be detrimental. For example, Sun et al. found that excessive autophagy leads to cell death, suggesting that brain tissue benefits from a controlled autophagic response (Jin et al., 2019). Identifying pathways that can restore a balanced autophagy system will be critical in developing treatments for nervous system disorders and injuries.

## 3. Neural cell’s oxidative stress and melatonin

The multiple roles of melatonin and its metabolites as potent direct radical scavengers and indirect antioxidants are particularly intriguing. Melatonin’s effectiveness in combating the harmful by-products of oxygen and nitrogen that can cause damage to essential molecules is greatly enhanced by its ability to neutralize the destructive molecules along with the metabolites generated during the scavenging process (Xie et al., 2016; Ni et al., 2018). Since the initial discovery of these remarkable properties about ten years ago, there has been a significant increase in the number of published research papers demonstrating the efficacy of melatonin in preventing oxidative damage (Puyal et al., 2009; Lim et al., 2016). Numerous experiments have examined the effects of free radical damage on the accumulation of molecular waste and subsequent cell loss in the brain. For example, melatonin has been shown to have beneficial effects on the brain in models of stroke, Alzheimer’s disease, Parkinson’s disease, and Huntington’s disease (Shi et al., 2017).

Melatonin, unlike other antioxidants, can readily cross all morphophysiological barriers, including the blood-brain barrier, and enter cells and subcellular compartments. Melatonin has shown potential in the treatment of neurodegenerative diseases, but more research is needed before it can be used in neurological clinics (Zhou et al., 2017).

Melatonin activates mRNA transcription for superoxide dismutase and increases the activities of antioxidant enzymes such as GSHPx, glutathione reductase, and glucose-6-phosphate dehydrogenase. This leads to an increase in antioxidant capacity (Sun et al., 2018). In addition, melatonin inhibits the pro-oxidant enzyme nitric oxide synthase, at least at certain sites. Both *in vivo* and *in vitro* studies have shown that melatonin reduces lipid peroxidation and oxidative damage to nuclear DNA. Although most of these benefits have been observed with pharmacological concentrations of melatonin, a few studies have demonstrated their physiological relevance (Reiter et al., 2015; Guleri and Tiwari, 2020).

Melatonin has been found to attenuate neurological damage caused by several factors, including porphyria (β-aminolevulinic acid), hyperbaric oxygen, glutamate excitotoxicity, ischemia-reperfusion injury, and several neurotoxins when administered prophylactically. These findings suggest that the decline in endogenous melatonin levels with age may contribute to the onset or severity of age-related neurodegenerative disorders. In addition, melatonin has demonstrated its ability to reduce oxidative damage in several models of Parkinson’s disease, such as the auto-oxidation of dopamine and 1-methyl-4-phenyl-1 (Vázquez et al., 2017; Cardinali, 2019; Franco et al., 2022).

## 4. Inflammatory responses and melatonin

The inflammatory process after acute ischemic stroke occurs in three distinct phases (Raza and Naureen, 2020). The first phase is characterized by an immediate acute post-stroke response with clearance of necrotic cells by microglia/macrophages and an initial infiltration of leukocytes, mainly neutrophils. Microglia/macrophage polarization can vary depending on the stage of inflammation, specific microenvironmental cues, and other factors. Both M1 and M2 polarization play distinct roles in the immune response, with M1 polarization associated with pro-inflammatory functions and M2 polarization associated with anti-inflammatory and tissue repair functions. The second phase occurs few days after the ischemic injury and represents a subacute phase associated with resolution of the inflammatory process. Finally, the late phase involves repair by astrocytes and microglia supported by inflammatory cells. The duration of the late phase can vary and may last for weeks or even months, depending on the individual and the extent of the stroke (Reiter, 1998; Raza and Naureen, 2020).

Numerous cytokines can either initiate or suppress inflammatory responses. For example, when neurons die, they release DAMPs or “danger signals” that activate TLR4 in microglia, which triggers the production of inflammatory cytokines through the NF-κB pathway (Reiter, 1998). M1-polarized microglia produce pro-inflammatory cytokines such as IL-1, IL-6, IL-18, and TNF (Reiter, 1998). In addition, perivascular macrophages located between the brain surface and the endothelium produce chemokines, ROS, and pro-inflammatory cytokines such as IL-1, IL-12, IL-2, and TNF-α (Tchekalarova and Tzoneva, 2023). Once in the brain parenchyma, T helper cells damage neurons, and the neurovascular unit by generating ROS, IFN-γ, TNF-α, IL-1, IL-17, and IL-21 (Tchekalarova and Tzoneva, 2023), while natural killer T cells exert neurotoxic effects by releasing IL-2 and TNF-α (Reiter, 1998).

Most investigators have observed that IL-1β increases significantly (up to 40- to 60-fold) in the brain within 24 h after stroke onset (Lambertsen et al., 2012), although there are some conflicting views (Reiter et al., 2001). After an ischemic insult, IL-1 exacerbates brain damage and contributes significantly to the breakdown of the blood-brain barrier (BBB). Experimental studies showed that rats exposed to IL-1 had more severe brain damage (Gupta et al., 2003), While IL-1β-deficient mice exhibited smaller-volume infarcts compared to wild-type mice (Drieu et al., 2018). In addition, blood levels of TNF-α, IL-6, and IL-10 were found to be reduced, and patients treated with recombinant human IL-1 receptor antagonist (IL-1Ra or anakinra) had better clinical outcomes than those treated with placebo (Yang et al., 2019).

While melatonin is generally thought to have beneficial effects in most conditions, its specific role in certain situations remains controversial. For example, in a model of rheumatoid arthritis, melatonin was reported to have pro-inflammatory effects. It increased the production of IL-1 and IL-6 and exacerbated pain in joint damage (Iadecola and Anrather, 2011). According to Radogna et al. melatonin may have a pro-inflammatory function in acute inflammation by protecting leukocytes from cell death processes, thereby prolonging their lifespan. Once leukocytes have accumulated in the injured area, melatonin continues to stimulate the production of various inflammatory proteins. Melatonin has been shown to play a role in the survival of immune cells. Previous studies have shown that melatonin regulates the apoptotic process by balancing the anti-apoptotic protein B-cell lymphoma 2 (Bcl-2) and the pro-apoptotic protein Bcl-2-associated X (Bax) (Rayasam et al., 2018). It reduces Bax activity at the outer mitochondrial membrane, preventing cytochrome c release and reducing apoptosis. In addition, Espino and colleagues demonstrated that melatonin inhibits apoptosis of human leukocytes by scavenging free radicals, as indicated by reduced DNA fragmentation. These findings support the idea that melatonin may induce an inflammatory response by extending the lifespan of leukocytes in injured tissues. However, the molecular intricacies underlying the pro-inflammatory role of melatonin in specific diseases require further research.

**FIGURE 1:**
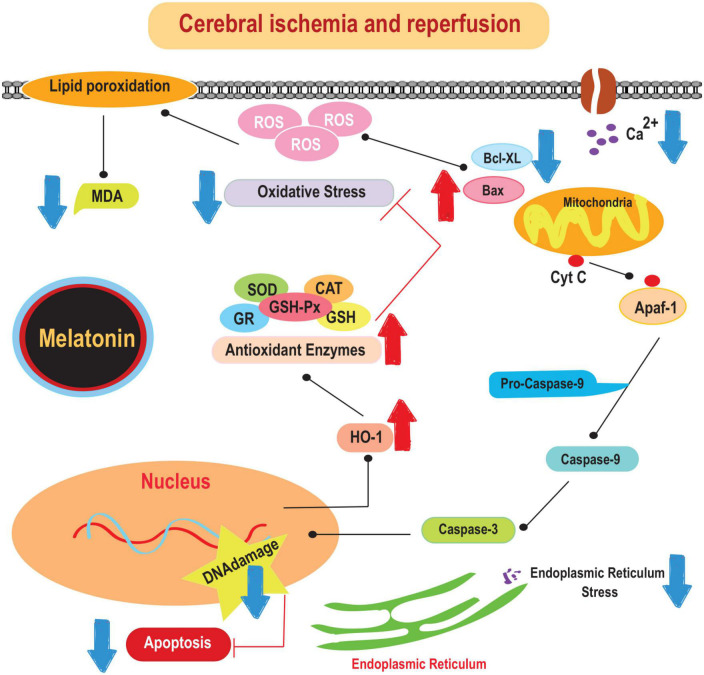
The mechanisms underlying the protective effects of melatonin on brain damage caused by cerebral-IRI. Cyt c, cytochrome c; SOD, superoxide dismutase; CAT, catalase; MDA, malonaldehyde; HO-1, heme oxygenase-1.

The beneficial effects of melatonin in the prevention of ischemic stroke have been recognized for several decades. For example, it has been shown to prevent oxidative stress, excitotoxicity, and mitochondrial dysfunction. It has been shown to reduce antioxidant stress, counteract excitotoxicity, and attenuate mitochondrial dysfunction. In addition, melatonin has anti-inflammatory properties (Emsley et al., 2007). Studies have shown that melatonin effectively reduces levels of pro-inflammatory cytokines such as IL-1 and TNF-α in both the cortex and hippocampus after middle cerebral artery occlusion (MCAO) (Tajalli-Nezhad et al., 2023). In addition, melatonin is responsible for attenuating the activation of microglial and astrocyte cells, as indicated by decreased production of markers such as ionized calcium-binding adapter molecule 1 (Iba-1) and glial fibrillary acidic protein (GFAP) (Boutin et al., 2001; Jurcau and Simion, 2022). This hormone directly modulates microglial activity by binding to its receptors on the microglial membrane and regulating the production of pro-inflammatory cytokines (Boutin et al., 2001). Therefore, melatonin may exert its anti-inflammatory properties in the context of ischemic stroke by modulating glial cell activation and cytokine production. The mechanisms underlying the protective effects of melatonin on brain damage caused by cerebral-IRI are illustrated in Figure 1.

## 5. Neuroprotective signaling pathways related to melatonin

Several neurological disorders, referred to as neurodegenerative diseases, are characterized by pathological changes, clinical manifestations, progressive deterioration and significant cellular dysfunction in brain networks. The biological response involved in synaptic transmission relies on second messengers activated by ligand-receptor interactions. By fine-tuning critical molecular signaling systems, we can gain insight into the underlying mechanisms of neurodegenerative disorders, with potential implications for preventive interventions in human health. Melatonin, primarily produced by the pineal gland, is thought to play a critical role in modulating these activities (Smith et al., 2018). It acts as an endogenous regulator of neurodegeneration and brain aging (Jiménez-Caliani et al., 2005; Radogna et al., 2008; Andrabi et al., 2015). The use of melatonin in clinical settings for the treatment of neurological disorders is promising given its minimal known adverse effects, as reduced melatonin production has been implicated in the etiology of Alzheimer’s disease (AD), Parkinson’s disease (PD), and Huntington’s disease (HD) (Rancan et al., 2018). Research studies have documented melatonin’s ability to prevent neurodegenerative pathology. These studies suggest that melatonin improves cognition and regulates neuroplasticity to mitigate neurodegenerative processes (Azedi et al., 2019).

### 5.1. Melatonin and Wnt/β-catenin signaling

The Wnt/β-catenin signaling pathway plays a major role in adult neurogenesis, controlling processes such as synaptogenesis, cell migration, cell fate determination, and integration into neuronal circuits (Pei and Cheung, 2004). Activation of β-catenin, a key component of this pathway, increases the population of neural progenitor cells. Wnt/β-catenin signaling is critical for the proliferation of neural stem cells (NSCs) by regulating the precise timing and cellular localization, including migration, of these cells (Shukla et al., 2019). β-Catenin regulates the proliferation and differentiation of neural progenitors during mammalian neuronal development, ultimately influencing the size of the cerebral cortex (Srinivasan et al., 2006). This suggests that Wnt signaling is involved in synaptic plasticity, memory formation, and cognitive function during aging. Notably, melatonin has been found to protect neurons by activating β-catenin signaling and regulating mitochondrial membrane potential to prevent apoptosis. which has been observed in a rat model of spinal cord injury (Pandi-Perumal et al., 2013; Shukla et al., 2017). In addition, melatonin promotes the generation of progenitor cells in the adult rat hippocampus and adult mouse subventricular zone through its interaction with the melatonin receptor MT1, which cooperates with growth factors to activate the ERK/MAPK pathway (Alghamdi, 2018; Corpas et al., 2018).

Transfection of β-catenin provides evidence for the importance of repairing the damaged brain and promotes the maintenance of neurogenesis, particularly the generation of hippocampal progenitor cells (Oliva et al., 2018). In a mouse model of AD, activation of Wnt signaling has been shown to reduce amyloidosis by decreasing APP cleavage and A formation (Pöschl et al., 2013). Melatonin enhances its mechanistic neuroprotection against Alzheimer’s disease (AD) through its beneficial effects on GSK3 and its ability to enhance the disruption of β-catenin expression (Chenn and Walsh, 2002). In a Drosophila model of Parkinson’s disease (PD), Wnt signaling is essential for protecting dopamine-producing neurons during disease progression (Jeong and Park, 2015). Melatonin treatment increases the expression of glial cell-derived neurotrophic factor mRNA, which improves the survival of neural stem cells (NSCs) and dopaminergic neurons, thereby promoting neuronal survival. In addition, ectopic production of β-catenin contributes to the attenuation of rotenone-induced dyskinesia in Parkinson’s disease (Shen et al., 2017).

### 5.2. Melatonin and STAT signaling pathway

Recent studies have investigated the role of melatonin in various signaling pathways, including the JAK-STAT pathway (Sotthibundhu et al., 2016). The STAT signaling system plays a critical role in cell proliferation, differentiation, cell death, and immune regulation. It is also essential for the signal transduction of many cytokines (Tocharus et al., 2014). In response to IL-4 and IL-13, STAT6 can be activated to perform cytoplasmic functions that modify the transcription factor called GATA3 (GATA binding protein 3). Activation of STAT6 by IL-4 and IL-13 leads to its phosphorylation, allowing it to form homodimers and translocate to the nucleus. Once in the nucleus, STAT6 interacts with GATA3 and induces its transcriptional activity. GATA3 is a key transcription factor involved in the differentiation and function of T helper 2 (Th2) cells, which play a critical role in immune responses against parasites and allergic diseases (Vallée and Lecarpentier, 2016). Upon phosphorylation of the critical tyrosine residue in STAT6, it dimerizes, translocates from the cytoplasm to the nucleus, and upregulates the transcription of specific target genes (Tocharus et al., 2014). Research has shown that IL-4-STAT6 signaling, through direct transcriptional regulation of inflammatory enhancers, reduces the sensitivity of macrophages to endogenous signals associated with pathogens, stress, and injury (Vallée and Lecarpentier, 2016).

Hyperglycemia has been shown to induce MCP-1 and iNOS expression, decrease Arg-1 and CD206 expression, increase STAT1 activation, and decrease STAT6 activity in intrahepatic macrophages isolated from APAP-exposed livers, while inhibiting M2 polarization (Huang et al., 2018). One study found that miR-449c-5p targets STAT6 and suppresses its expression. Consistent with this finding, SNHG4 overexpression increased the production of Arg-1 and CD206, enhanced STAT6 phosphorylation, and promoted the M1-to-M2 transition of microglia in the presence of ODG. Furthermore, the study observed a significant increase in the production of anti-inflammatory molecules, including IL-4, IL-10, and TGF-β, which effectively reduced the detrimental effects of inflammation on neurons (Shukla et al., 2017).

The JAK-STAT signaling pathway plays a role in mitigating the initial brain damage following subarachnoid hemorrhage (SAH), and melatonin has been shown to have a beneficial effect in this regard. In a rat study, melatonin was shown to improve neurological function and reduce neuronal apoptosis (cell death) and brain edema within 24 h of SAH (Huang et al., 2018). The JAK1/STAT3 pathway was implicated in this mechanism, and the beneficial effects of melatonin were partially diminished when a JAK1 inhibitor was administered, indicating the importance of this pathway in mediating the effects of melatonin (Shukla et al., 2017).

### 5.3. Melatonin and Notch signaling pathway

The Notch signaling pathway is a well conserved signaling pathway that controls cell growth, cell differentiation, and cell fate specification in both embryonic and adult stages (Lendahl and Siebel, 2017). The relationship between melatonin and the Notch signaling pathway has been investigated in several studies (Stephano et al., 2018). In the context of intrauterine growth restriction (IUGR) in rats, melatonin appears to have a potential modulatory role in the Notch pathway. The Notch signaling pathway plays a critical role in cell proliferation, differentiation, and programmed cell death, and its activity may affect fetal development and placental function. In IUGR placentas, upregulation of Notch receptors, ligands, and enzymes involved in processing and cleavage of Notch proteins was observed. Melatonin has been investigated in several studies as a potential intervention to rescue IUGR placentas. However, it is worth noting that one particular study indicated that melatonin treatment did not rescue fetal body weight or placental weight (Shukla et al., 2019; Tang et al., 2019).

Melatonin has been shown to have inhibitory effects on glioblastoma stem-like cells (GSCs) by targeting the EZH2-NOTCH1 signaling axis. GSCs are known to play a critical role in glioma growth and in conferring resistance to radiation and chemotherapy, leading to tumor recurrence (Chase and Cross, 2011). Melatonin has been shown to significantly reduce the viability and self-renewal capacity of GSCs, accompanied by a decrease in the expression of stem cell markers (Shukla et al., 2017). The EZH2-NOTCH1 signaling pathway was identified as the key mechanism through which melatonin exerted its effects on GSCs (Stephano et al., 2018). Melatonin partially suppressed GSC properties by modulating the EZH2-NOTCH1 signaling axis, highlighting its potential as a therapeutic agent in targeting GSCs (Shukla et al., 2019).

Melatonin promotes the transcription of metallopeptidase Domain 10 (ADAM10) (Lawrence and Natoli, 2011; Tang et al., 2019). On the other hand, melatonin controls the amyloidogenic processing of APP by inhibiting overexpression of beta-site amyloid precursor protein cleaving enzyme (BACE1) and modulation of presenilin-1 (Hou et al., 1994). In addition, melatonin inhibits the profibrillogenic effect of APOE4 on A-peptides (Yang et al., 2018). These modes of action suggest that melatonin can control Notch signaling by acting on various targets. The effects of Aβ-42 on Notch1, NICD, hairy, and Enhancer of Split 1 (Hes1) and Musashi-1 are also enhanced by melatonin (Wang Q. et al., 2019). Changes in neurogenesis are followed by decreased expression of Notch1, NICD, and Hes5 in the context of the pathogenesis of PD, suggesting that accumulation of–synuclein may impede nasopharyngeal carcinoma (NPC) survival by impairing the Notch signaling system (Zhang et al., 2020). Furthermore, melatonin controls Leucine-rich repeat kinase 2 (LRRK2), an essential protein associated with the pathogenesis of familial and idiopathic PD, and alteration of Notch signaling is an element that contributes to the etiology of the disease associated with LRRK2, as demonstrated in mature neurons (Li et al., 2019b). New loci relevant to neurodegenerative diseases could be discovered through epigenome-wide research, which would be particularly helpful for those still in the early stages of the disease spectrum.

## 6. Target of melatonin in neuroprotection under (I/R) injury

The human pineal gland, as well as many animals, produces the amine hormone melatonin, which acts as a free radical scavenger (Polychronidou et al., 2015). Melatonin has previously shown various pharmacological effects, including the ability to reduce oxidative stress, regulate circadian rhythms, prevent apoptosis, and reduce inflammation (Lendahl and Siebel, 2017; Lin et al., 2018). The receptor-dependent signaling action of melatonin and its direct antioxidant action can both be used to mediate its therapeutic effects. Several lines of evidence suggest that the melatonergic system plays a role in several neurodegenerative diseases. In the acute phase of stroke, melatonin administration has been shown to have several beneficial effects. It reduces the size of the ischemic infarct, decreases DNA fragmentation, inhibits the release of mitochondrial cytochrome c, and inhibits caspase-3 activity in the rat brain (Lee et al., 2007). Melatonin also reduces the cellular inflammatory response in the brain (Chase and Cross, 2011) and protects against oxidative damage. In addition, it provides protection against gray and white matter damage, improves neuroplasticity, and improves neurobehavioral and electrophysiological outcomes in rats following middle cerebral artery (MCA) occlusion (Lee et al., 2007). In recent cases, melatonin is critical to reducing IR injury, especially cerebral I/R injury (Kim et al., 2015; Zhang et al., 2016; Wang et al., 2020). Melatonin has been shown to have a variety of pharmacological functions in protecting against ischemic brain injury. These functions include regulating circadian rhythm, exerting antioxidant effects, reducing inflammation, and inhibiting apoptosis (Saberi et al., 2019). Studies have reported that melatonin treatment affects the expression of matrix metalloproteinase-9 (MMP-9) and MMP-13, while reducing the expression of transforming growth factor-beta (TGF-β), resulting in the inhibition of fibrosis (Yoon et al., 2020). Furthermore, melatonin-stimulated exosomes derived from mesenchymal stem cells (MSCs) were found to enhance functional recovery in acute liver ischemia-reperfusion injury, suggesting that melatonin modifies the content of exosomes, which in turn modulates the microenvironment through paracrine mechanisms (Manchester et al., 2015). Downregulation of exosomal miR-100-5p and miR-199a-5p, which directly target toll-like receptor 4 (TLR4), was identified as one of the regulatory effects of melatonin-treated exosomal miRNAs (Saberi et al., 2019). According to a growing body of research, melatonin reduces oxidative stress in cells, decreases excess calcium in cells, prevents endoplasmic stress, and prevents mitochondrial apoptosis (DeGracia and Montie, 2004; Fernández et al., 2015). Administration of melatonin to streptozotocin-induced diabetic mice and high glucose-treated HT22 cells was found to improve neurological impairment, reduce brain infarct size and brain edema, and improve cell viability. In addition, melatonin treatment reduced mitochondrial swelling, ROS production, and cytoplasmic cytochrome c release and increased mitochondrial antioxidant enzyme activity, adenosine triphosphate production, and mitochondrial membrane potential. Furthermore, by improving mitochondrial damage and stimulating Akt-Sirtuin 3 (SIRT3)-Superoxide dismutase-2 (SOD2) signaling, melatonin Cerebral-IR reduced damage in diabetic mice (DeGracia and Montie, 2004).

In the ischemic brain, oxidative stress and the generation of reactive oxygen species (ROS) can lead to oxidative damage in endoplasmic reticulum (ER) organelles, contributing to neuronal cell death after ischemia-reperfusion injury. ER stress triggers the activation of the PERK pathway, which phosphorylates eIF2α, suppresses protein synthesis, and promotes translation of the transcription factors ATF4 and CHOP. This ultimately leads to the activation of cell death signals (Lange et al., 2008). Studies have shown that mice lacking ATF4 have smaller infarcts, improved behavioral outcomes, and increased resistance to neuronal cell death in the context of ischemic stroke, highlighting the pro-death role of increased ATF4 expression (Chen et al., 2009).

Following ischemia-reperfusion, melatonin has been shown to reduce neuronal cell death in the ischemic brain through its potent free radical scavenging and antioxidant effects (Lee et al., 2005; Lin et al., 2018). Melatonin’s ability to counteract oxidative stress and scavenge free radicals contributes to its neuroprotective properties in ischemic conditions.

Endoplasmic reticulum stress plays an important role in mediating brain ischemia-reperfusion injury (Wu et al., 2013). Research has shown that ischemia-reperfusion injury leads to increased expression of ER stress-associated proteins such as p-PERK, p-eIF2α, ATF4, and CHOP in the penumbra and ischemic cortex (Lee et al., 2009). However, melatonin treatment was found to significantly reduce the expression of p-PERK, p-eIF2α, ATF4, and CHOP in both rat brain and cultured neurons after ischemia-reperfusion injury. Furthermore, melatonin treatment has been shown to reduce the levels of apoptosis markers, including cytochrome c and cleaved caspase-3, resulting from ischemia-reperfusion injury (Wu et al., 2013). These findings highlight the potential of melatonin to attenuate ER stress and mitigate neuronal damage associated with ischemia-reperfusion injury. Pretreatment with melatonin was shown to inhibit the expression of p-PERK and p-eIF2α in a dose-dependent manner. The optimal dose of melatonin for inhibition of p-PERK and p-eIF2α in cultured neurons subjected to oxygen-glucose deprivation (OGD) was found to be between 20–50 μM (Wu et al., 2013). Consistent with previous studies (Tai et al., 2011), a melatonin dose at 20–50 μM showed the best radical scavenging performance as detected by DPPH and ATBS assay. However, further investigation requires further investigation of the underlying biochemical and molecular processes by which melatonin exerts its neuroprotective effects in cerebral I/R injury. Its functions appear diverse and may have the power to influence various aspects of human health.

## 7. Conclusion

Melatonin, a naturally occurring hormone that follows circadian rhythms, has emerged as a promising therapy for ischemic stroke by mitigating the adverse effects of ischemia-reperfusion injury. Its cerebroprotective mechanisms include antioxidant properties, anti-inflammatory effects, preservation of mitochondrial function, and regulation of vascular tone. Melatonin acts as an effective scavenger of reactive oxygen species, modulates the immune response, protects mitochondrial integrity, and improves blood flow. These mechanisms contribute to minimizing tissue damage and improving outcomes in ischemic stroke, highlighting the potential of melatonin as a therapeutic agent. Further research is needed to optimize the therapeutic use of melatonin in this setting, given its demonstrated efficacy and safety profile.

## Author contributions

MT and BS draw the figures. MT, AN, HY, and GD wrote the manuscript. All authors revised the manuscript and approved the submitted version.

## References

[B1] Abolhasanpour N. Alihosseini S. Golipourkhalili S. Badalzadeh R. Mahmoudi J. Hosseini L. (2021). Effect of melatonin on endoplasmic reticulum-mitochondrial crosstalk in stroke. *Arch. Med. Res.* 52 673–682. 10.1016/j.arcmed.2021.04.002 33926763

[B2] Ajoolabady A. Shademan B. Avci C. B. Nikanfar M. Nourazarian A. Laghousi D. (2022). Diagnostic potential of autophagy-5 protein, apolipoprotein B-48, and oxidative stress markers in serum of patients with early-stage ischemic stroke. *World Neurosurg.* 167 e656–e663. 10.1016/j.wneu.2022.08.063 36030010

[B3] Alghamdi B. S. (2018). The neuroprotective role of melatonin in neurological disorders. *J. Neurosci. Res.* 96 1136–1149. 10.1002/jnr.24220 29498103 PMC6001545

[B4] Amanakis G. Kleinbongard P. Heusch G. Skyschally A. (2019). Attenuation of ST-segment elevation after ischemic conditioning maneuvers reflects cardioprotection online. *Basic Res. Cardiol.* 114:22. 10.1007/s00395-019-0732-3 30937537

[B5] Andrabi S. S. Parvez S. Tabassum H. (2015). Melatonin and ischemic stroke: Mechanistic roles and action. *Adv. Pharm. Sci.* 2015:384750. 10.1155/2015/384750 26435711 PMC4575994

[B6] Angelova P. R. Myers I. Abramov A. Y. (2023). Carbon monoxide neurotoxicity is triggered by oxidative stress induced by ROS production from three distinct cellular sources. *Redox Biol.* 7:102598. 10.1016/j.redox.2022.102598 36640724 PMC9852609

[B7] Antonucci L. Fagman J. B. Kim J. Y. Todoric J. Gukovsky I. Mackey M. (2015). Basal autophagy maintains pancreatic acinar cell homeostasis and protein synthesis and prevents ER stress. *Proc. Natl. Acad. Sci. U.S.A.* 112 E6166–E6174. 10.1073/pnas.1519384112 26512112 PMC4653219

[B8] Atanassova P. A Terzieva D. D. Dimitrov B. D. (2009). Impaired nocturnal melatonin in acute phase of ischaemic stroke: Cross-sectional matched case–control analysis. *J. neuroendocrinol.* 21 657–663. 10.1111/j.1365-2826.2009.01881.x 19453822

[B9] Azedi F. Mehrpour M. Talebi S. Zendedel A. Kazemnejad S. Mousavizadeh K. (2019). Melatonin regulates neuroinflammation ischemic stroke damage through interactions with microglia in reperfusion phase. *Brain Res.* 1723:146401. 10.1016/j.brainres.2019.146401 31445031

[B10] Birnbaum Y. Tran D. Bajaj M. Ye Y. (2019). DPP-4 inhibition by linagliptin prevents cardiac dysfunction and inflammation by targeting the Nlrp3/ASC inflammasome. *Basic Res. Cardiol.* 114 1–22. 10.1007/s00395-019-0743-0 31388770

[B11] Boutin H. LeFeuvre R. A. Horai R. Asano M. Iwakura Y. Rothwell N. J. (2001). Role of IL-1alpha and IL-1beta in ischemic brain damage. *J. Neurosci.* 21 5528–5534. 10.1523/JNEUROSCI.21-15-05528.2001 11466424 PMC6762680

[B12] Bussi C. Peralta Ramos J. M. Arroyo D. S. Gaviglio E. A. Gallea J. I. Wang J. M. (2017). Autophagy down regulates pro-inflammatory mediators in BV2 microglial cells and rescues both LPS and alpha-synuclein induced neuronal cell death. *Sci. Rep.* 7:43153. 10.1038/srep43153 28256519 PMC5335665

[B13] Caplan L. R. Simon R. P. Hassani S. (2023). “Chapter 27 - Cerebrovascular disease—stroke,” in *Neurobiology of brain disorders*, 2nd Edn, eds Zigmond M. J. Wiley C. A. Chesselet M.-F. (Cambridge, MA: Academic Press), 457–476. 10.1016/B978-0-323-85654-6.00044-7

[B14] Cardinali D. P. (2019). Melatonin: Clinical perspectives in neurodegeneration. *Front. Endocrinol.* 10:480. 10.3389/fendo.2019.00480 31379746 PMC6646522

[B15] Chase A. Cross N. C. (2011). Aberrations of EZH2 in cancer. *Clin. Cancer Res.* 17 2613–2618. 10.1158/1078-0432.CCR-10-2156 21367748

[B16] Chen H. Y. Hung Y. C. Chen T. Y. Huang S. Y. Wang Y. H. Lee W. T. (2009). Melatonin improves presynaptic protein, SNAP-25, expression and dendritic spine density and enhances functional and electrophysiological recovery following transient focal cerebral ischemia in rats. *J. Pineal Res.* 47 260–270. 10.1111/j.1600-079X.2009.00709.x 19709397

[B17] Chenn A. Walsh C. A. (2002). Regulation of cerebral cortical size by control of cell cycle exit in neural precursors. *Science* 297 365–369. 10.1126/science.1074192 12130776

[B18] Corpas R. Griñán-Ferré C. Palomera-Ávalos V. Porquet D. García de Frutos P. Franciscato Cozzolino S. M. (2018). Melatonin induces mechanisms of brain resilience against neurodegeneration. *J. Pineal Res.* 65:e12515. 10.1111/jpi.12515 29907977

[B19] Cummins P. M. (2011). Occludin: One protein, many forms. *Mol. Cell. Biol.* 32 242–250. 10.1128/MCB.06029-11 22083955 PMC3255790

[B20] DeGracia D. J. Montie H. L. (2004). Cerebral ischemia and the unfolded protein response. *J. Neurochem.* 91 1–8. 10.1111/j.1471-4159.2004.02703.x 15379881

[B21] Deng H. Zuo X. Zhang J. Liu X. Liu L. Xu Q. (2015). α−lipoic acid protects against cerebral ischemia/reperfusion−induced injury in rats. *Mol. Med. Rep.* 11 3659–3665. 10.3892/mmr.2015.3170 25572614

[B22] Deussen A. (2018). Mechanisms underlying coronary autoregulation continue to await clarification. *Basic Res. Cardiol.* 113:34. 10.1007/s00395-018-0693-y 30074094

[B23] Drieu A. Levard D. Vivien D. Rubio M. (2018). Anti-inflammatory treatments for stroke: From bench to bedside. *Ther. Adv. Neurol. Dis.* 11 1–15. 10.1177/1756286418789854 30083232 PMC6066814

[B24] Ebrahimi N. D. Shojaei-Zarghani S. Taherifard E. Dastghaib S. Parsa S. Mohammadi N. (2023). Protective effects of melatonin against physical injuries to testicular tissue: A systematic review and meta-analysis of animal models. *Front. Endocrinol.* 14:1123999. 10.3389/fendo.2023.1123999 36798664 PMC9927015

[B25] Emsley H. C. A. Smith C. J. Gavin C. M. Georgiou R. F. Vail A. Barberan M. E. (2007). Clinical outcome following acute ischaemic stroke relates to both activation and autoregulatory inhibition of cytokine production. *BMC Neurol.* 7:5. 10.1186/1471-2377-7-5 17328808 PMC1810309

[B26] Fernández A. Ordóñez R. Reiter R. J. González-Gallego J. Mauriz J. L. (2015). Melatonin and endoplasmic reticulum stress: Relation to autophagy and apoptosis. *J. Pineal Res.* 59 292–307. 10.1111/jpi.12264 26201382

[B27] Franco P. I. do Carmo Neto J. R. Milhomem A. C. Machado J. R. Miguel M. P. (2022). Antitumor effect of melatonin on breast cancer in experimental models: A systematic review. *Biochim. Biophys. Acta Rev. Cancer* 1878:188838. 10.1016/j.bbcan.2022.188838 36403922

[B28] Gao X. M. Su Y. Moore S. Han L. P. Kiriazis H. Lu Q. (2019). Relaxin mitigates microvascular damage and inflammation following cardiac ischemia–reperfusion. *Basic Res. Cardiol.* 114:30. 10.1007/s00395-019-0739-9 31218471

[B29] Guleri S. Tiwari A. (2020). “Algae and ageing,” in *Microalgae biotechnology for food, health and high value products*, eds Alam M. A. Xu J. L. Wang Z. (Singapore: Springer), 267–293. 10.1007/978-981-15-0169-2_8

[B30] Gupta Y. K. Gupta M. Kohli K. (2003). Neuroprotective role of melatonin in oxidative stress vulnerable brain. *Indian J. Physiol. Pharmacol.* 47 373–386. 15266948

[B31] Hou J. Schindler U. Henzel W. J. Ho T. C. Brasseur M. McKnight S. L. (1994). An interleukin-4-induced transcription factor: IL-4 Stat. *Science* 265 1701–1706. 10.1126/science.8085155 8085155

[B32] Hu X. Leak R. K. Shi Y. Suenaga J. Gao Y. Zheng P. (2015). Microglial and macrophage polarization—new prospects for brain repair. *Nat. Rev. Neurol.* 11 56–64. 10.1038/nrneurol.2014.207 25385337 PMC4395497

[B33] Huang M. Liang Y. Chen H. Xu B. Chai C. Xing P. (2018). The role of fluoxetine in activating Wnt/β-catenin signaling and repressing β-amyloid production in an Alzheimer mouse model. *Front. Aging Neurosci.* 10:164. 10.3389/fnagi.2018.00164 29910725 PMC5992518

[B34] Iadecola C. (2017). The neurovascular unit coming of age: A journey through neurovascular coupling in health and disease. *Neuron* 96 17–42. 10.1016/j.neuron.2017.07.030 28957666 PMC5657612

[B35] Iadecola C. Anrather J. (2011). The immunology of stroke: From mechanisms to translation. *Nat. Med.* 17 796–808. 10.1038/nm.2399 21738161 PMC3137275

[B36] Jeong J. K. Park S. Y. (2015). Melatonin regulates the autophagic flux via activation of alpha-7 nicotinic acetylcholine receptors. *J. Pineal Res.* 59 24–37. 10.1111/jpi.12235 25808024

[B37] Jiménez-Caliani A. J. Jiménez-Jorge S. Molinero P. Guerrero J. Fernández-Santos J. Martín-Lacave I. (2005). Dual effect of melatonin as proinflammatory and antioxidant in collagen-induced arthritis in rats. *J. Pineal Res.* 38 93–99. 10.1111/j.1600-079X.2004.00175.x 15683463

[B38] Jin J. Sun H. Liu D. Wang H. Liu Q. Chen H. (2019). LRG1 promotes apoptosis and autophagy through the TGFβ-smad1/5 signaling pathway to exacerbate ischemia/reperfusion injury. *Neuroscience* 413 123–134. 10.1016/j.neuroscience.2019.06.008 31220542

[B39] Jolivel V. Bicker F. Binamé F. Ploen R. Keller S. Gollan R. (2015). Perivascular microglia promote blood vessel disintegration in the ischemic penumbra. *Acta Neuropathol.* 129 279–295. 10.1007/s00401-014-1372-1 25500713

[B40] Jurcau A. Simion A. (2022). Neuroinflammation in cerebral ischemia and ischemia/reperfusion injuries: From pathophysiology to therapeutic strategies. *Int. J. Mol. Sci.* 23:14. 10.3390/ijms23010014 35008440 PMC8744548

[B41] Kalogeris T. Baines C. P. Krenz M. Korthuis R. J. (2016). Ischemia/Reperfusion. *Compr. Physiol.* 7 113–170. 10.1002/cphy.c160006 28135002 PMC5648017

[B42] Kim S. H. Joshi K. Ezhilarasan R. Myers T. R. Siu J. Gu C. (2015). EZH2 protects glioma stem cells from radiation-induced cell death in a MELK/FOXM1-dependent manner. *Stem Cell Rep.* 4 226–238. 10.1016/j.stemcr.2014.12.006 25601206 PMC4325196

[B43] Knowland D. Arac A. Sekiguchi K. J. Hsu M. Lutz S. E. Perrino J. (2014). Stepwise recruitment of transcellular and paracellular pathways underlies blood-brain barrier breakdown in stroke. *Neuron* 82 603–617. 10.1016/j.neuron.2014.03.003 24746419 PMC4016169

[B44] Kruk J. Aboul-Enein B. H. Duchnik E. Marchlewicz M. (2022). Antioxidative properties of phenolic compounds and their effect on oxidative stress induced by severe physical exercise. *J. Physiol. Sci.* 72:19. 10.1186/s12576-022-00845-1 35931969 PMC10717775

[B45] Kryl’skii E. D. Popova T. N. Safonova O. A. Stolyarova A. O. Razuvaev G. A. de Carvalho M. A. (2019). Transcriptional regulation of antioxidant enzymes activity and modulation of oxidative stress by melatonin in rats under cerebral ischemia/reperfusion conditions. *Neuroscience* 406 653–666. 10.1016/j.neuroscience.2019.01.046 30716363

[B46] Kuznetsov A. V. Javadov S. Margreiter R. Grimm M. Hagenbuchner J. Ausserlechner M. J. (2019). The role of mitochondria in the mechanisms of cardiac ischemia-reperfusion injury. *Antioxidants* 8:454. 10.3390/antiox8100454 31590423 PMC6826663

[B47] Lambertsen K. L. Biber K. Finsen B. (2012). Inflammatory cytokines in experimental and human stroke. *J. Cereb. Blood Flow Metab.* 32 1677–1698. 10.1038/jcbfm.2012.88 22739623 PMC3434626

[B48] Lange P. S. Chavez J. C. Pinto J. T Coppola G. Sun C. W. Townes T. M. (2008). ATF4 is an oxidative stress–inducible, prodeath transcription factor in neurons *in vitro* and *in vivo*. *J. Exp. Med.* 205 1227–1242. 10.1084/jem.20071460 18458112 PMC2373852

[B49] Lawrence T. Natoli G. (2011). Transcriptional regulation of macrophage polarization: Enabling diversity with identity. *Nat. Rev. Immunol.* 11 750–761. 10.1038/nri3088 22025054

[B50] Lee E. J. Chen H. Y. Hung Y. C. Chen T. Y. Lee M. Y Yu S. C. (2009). Therapeutic window for cinnamophilin following oxygen–glucose deprivation and transient focal cerebral ischemia. *Exp. Neurol.* 217 74–83. 10.1016/j.expneurol.2009.01.019 19416670

[B51] Lee E. J. Lee M. Y. Chen H. Y. Hsu Y. S. Wu T. S. Chen S. T. (2005). Melatonin attenuates gray and white matter damage in a mouse model of transient focal cerebral ischemia. *J. Pineal Res.* 38 42–52. 10.1111/j.1600-079X.2004.00173.x 15617536

[B52] Lee M. Y. Kuan Y. H. Chen H. Y. Chen T. Y. Chen S. T. Huang C. C. (2007). Intravenous administration of melatonin reduces the intracerebral cellular inflammatory response following transient focal cerebral ischemia in rats. *J. Pineal Res.* 42 297–309. 10.1111/j.1600-079X.2007.00420.x 17349029

[B53] Lendahl U. Siebel C. (2017). Notch signaling in development, tissue homeostasis, and disease. *Physiol. Rev.* 97 1235–1294. 10.1152/physrev.00005.2017 28794168

[B54] Li Q. Guo Z. Wu C. Tu Y. Wu Y. Xie E. (2022). Ischemia preconditioning alleviates ischemia/reperfusion injury-induced coronary no-reflow and contraction of microvascular pericytes in rats. *Microvasc. Res.* 142:104349. 10.1016/j.mvr.2022.104349 35240123

[B55] Li S. Jiang D. Rosenkrans Z. T. Barnhart T. E. Ehlerding E. B. Ni D. (2019a). Aptamer-conjugated framework nucleic acids for the repair of cerebral ischemia-reperfusion injury. *Nano Lett.* 19 7334–7341. 10.1021/acs.nanolett.9b02958 31518140 PMC6876547

[B56] Li S. Yang S. Sun B. Hang C. (2019b). Melatonin attenuates early brain injury after subarachnoid hemorrhage by the JAK-STAT signaling pathway. *Int. J. Clin. Exp. Pathol.* 12 909–915. 31933900 PMC6945149

[B57] Lim Y. Cho H. Kim E. K. (2016). Brain metabolism as a modulator of autophagy in neurodegeneration. *Brain Res.* 1649 158–165. 10.1016/j.brainres.2016.02.049 26970520

[B58] Lin H. C. Narasimhan P. Liu S. Y. Chan P. H. Lai I. R. (2015). Postconditioning mitigates cell death following oxygen and glucose deprivation in PC12 cells and forebrain reperfusion injury in rats. *J. Neurosci. Res.* 93 140–148. 10.1002/jnr.23460 25082329

[B59] Lin L. Wang X. Yu Z. (2016). Ischemia-reperfusion injury in the brain: Mechanisms and potential therapeutic strategies. *Biochem. Pharmacol.* 5:213. 10.4172/2167-0501.1000213 29888120 PMC5991620

[B60] Lin Y. W. Chen T. Y. Hung C. Y. Tai S. H. Huang S. Y. Chang C. C. (2018). Melatonin protects brain against ischemia/reperfusion injury by attenuating endoplasmic reticulum stress. *Int. J. Mol. Med.* 42 182–192. 10.3892/ijmm.2018.3607 29620280 PMC5979830

[B61] Liu L. Chen H. Jin J. Tang Z. Yin P. Zhong D. (2019). Melatonin ameliorates cerebral ischemia/reperfusion injury through SIRT3 activation. *Life Sci.* 239:117036. 10.1016/j.lfs.2019.117036 31697951

[B62] Liu N. B. Wu M. Chen C. Fujino M. Huang J. S. Zhu P. (2019). Novel molecular targets participating in myocardial ischemia-reperfusion injury and cardioprotection. *Cardiol. Res. Pract.* 8:2019. 10.1155/2019/6935147 31275641 PMC6558612

[B63] Liu Y. Tan Y. Cao G. Shi L. Song Y. Shan W. (2023a). Bergenin alleviates myocardial ischemia-reperfusion injury via SIRT1 signaling. *Biomed. Pharmacother.* 158:114100. 10.1016/j.biopha.2022.114100 36538860

[B64] Liu Y. Tan Y. Q. Zhou G. (2023b). Melatonin: A potential therapeutic approach for the management of primary Sjögren’s syndrome. *Immunol. Res.* 71 373–387. 10.1007/s12026-023-09360-w 36715831

[B65] Lorente L. Martín M. M. Pérez-Cejas A. Abreu-González P. Ramos L. Argueso M. (2016). Association between total antioxidant capacity and mortality in ischemic stroke patients. *Ann. Intens. Care.* 6:39. 10.1186/s13613-016-0143-7 27107565 PMC4842192

[B66] Lu S. Sun L. Shen J. Su F. Wang H. Ye Z. (2010). Protective effects of auricularia auricular polysaccharide on chronic cerebral ischemia injury in rats. *Chin. J. Pathophysiol.* 26 721–724.

[B67] Lucas S. M. Rothwell N. J. Gibson R. M. (2006). The role of inflammation in CNS injury and disease. *Br. J. Pharmacol.* 147 S232–S240. 10.1038/sj.bjp.0706400 16402109 PMC1760754

[B68] Magen S. Seybold H. Laloum D. Avin-Wittenberg T. (2022). Metabolism and autophagy in plants—a perfect match. *FEBS Lett.* 596 2133–2151. 10.1002/1873-3468.14359 35470431

[B69] Manchester L. C. Coto-Montes A. Boga J. A. Andersen L. P. Zhou Z. Galano A. (2015). Melatonin: An ancient molecule that makes oxygen metabolically tolerable. *J. Pineal Res.* 59 403–419. 10.1111/jpi.12267 26272235

[B70] Mao Z. J. Lin H. Xiao F. Y. Huang Z. Q. Chen Y. H. (2020). Melatonin against myocardial ischemia-reperfusion injury: A meta-analysis and mechanism insight from animal studies. *Oxid. Med. Cell. Longev.* 2020:1241065. 10.1155/2020/1241065 32685084 PMC7336233

[B71] McCartney P. J. Eteiba H. Maznyczka A. M. McEntegart M. Greenwood J. P. Muir D. F. (2019). Effect of low-dose intracoronary alteplase during primary percutaneous coronary intervention on microvascular obstruction in patients with acute myocardial infarction: A randomized clinical trial. *JAMA* 321 56–68. 10.1001/jama.2018.19802 30620371 PMC6583564

[B72] Moradi Majd R. Mayeli M. Rahmani F. (2020). Pathogenesis and promising therapeutics of Alzheimer disease through eIF2α pathway and correspondent kinases. *Metab. Brain Dis.* 35 1241–1250. 10.1007/s11011-020-00600-8 32681467

[B73] Mukherjee A. Sarkar S. Jana S. Swarnakar S. Das N. (2019). Neuro-protective role of nanocapsulated curcumin against cerebral ischemia-reperfusion induced oxidative injury. *Brain Res.* 1704 164–173. 10.1016/j.brainres.2018.10.016 30326199

[B74] Nazir S. A. McCann G. P. Greenwood J. P. Kunadian V. Khan J. N. Mahmoud I. Z. (2016). Strategies to attenuate micro-vascular obstruction during P-PCI: The randomized reperfusion facilitated by local adjunctive therapy in ST-elevation myocardial infarction trial. *Eur. Heart J.* 37 1910–1919. 10.1093/eurheartj/ehw136 27147610 PMC4917746

[B75] Neher J. J. Emmrich J. V. Fricker M. Mander P. K. Thery C. Brown G. C. (2013). Phagocytosis executes delayed neuronal death after focal brain ischemia. *Proc. Natl. Acad. Sci. U.S.A.* 110 E4098–E4107. 10.1073/pnas.1308679110 24101459 PMC3808587

[B76] Ni Y. Gu W. W. Liu Z. H. Zhu Y. M. Rong J. G. Kent T. A. (2018). RIP1K contributes to neuronal and astrocytic cell death in ischemic stroke via activating autophagic-lysosomal pathway. *Neuroscience* 371 60–74. 10.1016/j.neuroscience.2017.10.038 29102662

[B77] Oliva C. A. Montecinos-Oliva C. Inestrosa N. C. (2018). Wnt signaling in the central nervous system: New insights in health and disease. *Progr. Mol. Biol. Transl. Sci.* 153 81–130. 10.1016/bs.pmbts.2017.11.018 29389523

[B78] Orekhov A. N. Orekhova V. A. Nikiforov N. G. Myasoedova V. A. Grechko A. V. Romanenko E. B. (2019). Monocyte differentiation and macrophage polarization. *Vessel Plus* 3:10. 10.20517/2574-1209.2019.04

[B79] Pandi-Perumal S. R. BaHammam A. S. Brown G. M. Spence D. W. Bharti V. K. Kaur C. (2013). Melatonin antioxidative defense: Therapeutical implications for aging and neurodegenerative processes. *Neurotox. Res.* 23 267–300. 10.1007/s12640-012-9337-4 22739839

[B80] Patel A. R. Ritzel R. McCullough L. D. Liu F. (2013). Microglia, and ischemic stroke: A double-edged sword. *Int. J. Physiol. Pathophysiol. Pharmacol.* 5 73–90. 23750306 PMC3669736

[B81] Patel D. K. Strong J. (2019). The pleiotropic effects of sodium–glucose cotransporter-2 inhibitors: Beyond the glycemic benefit. *Diabetes Ther.* 10 1771–1792. 10.1007/s13300-019-00686-z 31456166 PMC6778563

[B82] Pei Z. Cheung R. T. (2004). Pretreatment with melatonin exerts antiinflammatory effects against ischemia/reperfusion injury in a rat middle cerebral artery occlusion stroke model. *J. Pineal Res.* 37 85–91. 10.1111/j.1600-079X.2004.00138.x 15298666

[B83] Pluta R. Furmaga-Jabłońska W. Januszewski S. Tarkowska A. (2023). Melatonin: A potential candidate for the treatment of experimental and clinical perinatal asphyxia. *Molecules* 28:1105. 10.3390/molecules28031105 36770769 PMC9919754

[B84] Polychronidou E. Vlachakis D. Vlamos P. Baumann M. Kossida S. (2015). “Notch signaling and ageing,” in *GeNeDis 2014: Neurodegeneration*, eds Vlamos P. Alexiou A. (Cham: Springer International Publishing), 25–36. 10.1007/978-3-319-08927-0_6 25416974

[B85] Pöschl J. Grammel D. Dorostkar M. M Kretzschmar H. A Schüller U. (2013). Constitutive activation of β-catenin in neural progenitors results in disrupted proliferation and migration of neurons within the central nervous system. *Dev. Biol.* 374 319–332. 10.1016/j.ydbio.2012.12.001 23237957

[B86] Puyal J. Ginet V. Vaslin A. Truttmann A. C. Clarke P. G. (2009). The two faces of autophagy in the nervous system. *Med. Sci.* 25 383–390.3. 10.1051/medsci/2009254383 19409191

[B87] Qian M. Fang X. Wang X. (2017). Autophagy and inflammation. *Clin Transl Med.* 6:24. 10.1186/s40169-017-0154-5 28748360 PMC5529308

[B88] Radogna F. Cristofanon S. Paternoster L. D’Alessio M. De Nicola M. Cerella C. (2008). Melatonin antagonizes the intrinsic pathway of apoptosis via mitochondrial targeting of bcl-2. *J. Pineal Res.* 44 316–325. 10.1111/j.1600-079X.2007.00532.x 18339127

[B89] Rahman M. M. Yang D. K. (2023). Melatonin supplement plus exercise effectively counteracts the challenges of isoproterenol-induced cardiac injury in rats. *Biomedicines* 11:428. 10.3390/biomedicines11020428 36830962 PMC9953439

[B90] Rancan L. Paredes S. D. García C. González P. Rodríguez-Bobada C. Calvo-Soto M. (2018). Comparison of the effect of melatonin treatment before and after brain ischemic injury in the inflammatory and apoptotic response in aged rats. *Int J Mol Sci.* 19:2097. 10.3390/ijms19072097 30029514 PMC6073988

[B91] Rayasam A. Hsu M. Kijak J. A. Kissel L. Hernandez G. Sandor M. (2018). Immune responses in stroke: How the immune system contributes to damage and healing after stroke and how this knowledge could be translated to better cures? *Immunology* 154 363–376. 10.1111/imm.12918 29494762 PMC6002204

[B92] Raza Z. Naureen Z. (2020). Melatonin ameliorates the drug induced nephrotoxicity: Molecular insights. *Nefrologia* 40 12–25. 10.1016/j.nefro.2019.06.009 31735377

[B93] Reiter R. J. (1998). Oxidative damage in the central nervous system: Protection by melatonin. *Progr. Neurobiol.* 56 359–384. 10.1016/S0301-0082(98)00052-5 9770244

[B94] Reiter R. J. Tan D. X. Manchester L. C. Calvo J. R. (2001). “Antioxidant capacity of melatonin,” in *Handbook of antioxidants*, (Boca Raton, FL: CRC Press), 584–633.

[B95] Reiter R. J. Tan D. X. Zhou Z. Cruz M. H. Fuentes-Broto L. Galano A. (2015). Phytomelatonin: Assisting plants to survive and thrive. *Molecules* 20 7396–7437. 10.3390/molecules20047396 25911967 PMC6272735

[B96] Rios-Navarro C. Marcos-Garces V. Bayes-Genis A. Husser O. Nuñez J. Bodi V. (2019). Microvascular obstruction in ST-segment elevation myocardial infarction: Looking back to move forward. Focus on CMR. *J. Clin. Med.* 8:1805. 10.3390/jcm8111805 31661823 PMC6912395

[B97] Saberi K. Pasbakhsh P. Omidi A. Borhani-Haghighi M. Nekoonam S. Omidi N. (2019). Melatonin preconditioning of bone marrow-derived mesenchymal stem cells promotes their engraftment and improves renal regeneration in a rat model of chronic kidney disease. *J. Mol. Histol.* 50 129–140. 10.1007/s10735-019-09812-4 30671880

[B98] Shademan B. Nourazarian A. Laghousi D. Karamad V. Nikanfar M. (2021). Exploring potential serum levels of Homocysteine, interleukin-1 beta, and apolipoprotein B 48 as new biomarkers for patients with ischemic stroke. *J. Clin. Lab. Anal.* 35:e23996. 10.1002/jcla.23996 34492129 PMC8551691

[B99] Sharma V. Mehdi M. M. (2023). Oxidative stress, inflammation and hormesis: The role of dietary and lifestyle modifications on aging. *Neurochem. Int.* 4:105490. 10.1016/j.neuint.2023.105490 36702401

[B100] Shen Z. Zhou Z. Gao S. Guo Y. Gao K. Wang H. (2017). Melatonin inhibits neural cell apoptosis and promotes locomotor recovery via activation of the Wnt/β-catenin signaling pathway after spinal cord injury. *Neurochem. Res.* 42 2336–2343. 10.1007/s11064-017-2251-7 28417262

[B101] Shi Z. Y. Deng J. X. Fu S. Wang L. Wang Q. Liu B. (2017). Protective effect of autophagy in neural ischemia and hypoxia: Negative regulation of the Wnt/β-catenin pathway. *Int. J. Mol. Med.* 40 1699–1708. 10.3892/ijmm.2017.3158 29039446 PMC5716434

[B102] Shin J. A. Lim S. M. Jeong S. I. Kang J. L. Park E. M. (2014). Noggin improves ischemic brain tissue repair and promotes alternative activation of microglia in mice. *Brain Behav. Immun.* 40 143–154. 10.1016/j.bbi.2014.03.013 24704569

[B103] Shukla M. Chinchalongporn V. Govitrapong P. Reiter R. J. (2019). The role of melatonin in targeting cell signaling pathways in neurodegeneration. *Ann. N. Y. Acad. Sci.* 1443 75–96. 10.1111/nyas.14005 30756405

[B104] Shukla M. Govitrapong P. Boontem P. Reiter R. J. Satayavivad J. (2017). Mechanisms of melatonin in alleviating Alzheimer’s disease. *Curr. Neuropharmacol.* 15 1010–1031. 10.2174/1570159X15666170313123454 28294066 PMC5652010

[B105] Smith C. J. Hulme S. Vail A. Heal C. Parry-Jones A. R. Scarth S. (2018). SCIL-STROKE (Subcutaneous interleukin-1 receptor antagonist in ischemic stroke): A randomized controlled phase 2 trial. *Stroke* 49 1210–1216. 10.1161/STROKEAHA.118.020750 29567761

[B106] Sotthibundhu A. Ekthuwapranee K. Govitrapong P. (2016). Comparison of melatonin with growth factors in promoting precursor cells proliferation in adult mouse subventricular zone. *EXCLI J.* 15:829. 10.17179/excli2016-606 PMC5341012 28275319

[B107] Srinivasan V. Pandi-Perumal S. R. Cardinali D. P. Poeggeler B. Hardeland R. (2006). Melatonin in Alzheimer’s disease and other neurodegenerative disorders. *Behav. Brain Funct.* 2 1–23. 10.1186/1744-9081-2-15 16674804 PMC1483829

[B108] Stephano F. Nolte S. Hoffmann J. El-Kholy S. von Frieling J. Bruchhaus I. (2018). Impaired Wnt signaling in dopamine containing neurons is associated with pathogenesis in a rotenone triggered Drosophila Parkinson’s disease model. *Sci. Rep.* 8:2372. 10.1038/s41598-018-20836-w 29403026 PMC5799194

[B109] Sun D. Wang W. Wang X. Wang Y. Xu X. Ping F. (2018). bFGF plays a neuroprotective role by suppressing excessive autophagy and apoptosis after transient global cerebral ischemia in rats. *Cell Death Dis.* 9:172. 10.1038/s41419-017-0229-7 29416039 PMC5833346

[B110] Suzen S. (2023). “Melatonin in aging and aging-related disorders,” in *Emerging anti-aging strategies*, ed. Rizvi S. I (Singapore: Springer), 155–189. 10.1007/978-981-19-7443-4_9

[B111] Tai S. H. Hung Y. C. Lee E. J. Lee A. C. Chen T. Y. Shen C. C (2011). Melatonin protects against transient focal cerebral ischemia in both reproductively active and estrogen-deficient female rats: The impact of circulating estrogen on its hormetic dose–response. *J. Pineal Res.* 50 292–303. 10.1111/j.1600-079X.2010.00839.x 21210839

[B112] Tajalli-Nezhad S. Mohammadi S. Atlasi M. A. Kheiran M. Moghadam S. E. Naderian H. (2023). Calcitriols modulate post-ischemic TLR signaling pathway in ischemic stroke patients. *J. Neuroimmunol.* 375:578013. 10.1016/j.jneuroim.2022.578013 36657372

[B113] Tang Y. Shen J. Zhang F. Yang F. Y. Liu M. (2019). Human serum albumin attenuates global cerebral ischemia/reperfusion-induced brain injury in a Wnt/β-Catenin/ROS signaling-dependent manner in rats. *Biomed. Pharmacother.* 115:108871. 10.1016/j.biopha.2019.108871 31026729

[B114] Tchekalarova J. Tzoneva R. (2023). Oxidative stress and aging as risk factors for Alzheimer’s disease and Parkinson’s disease: The role of the antioxidant melatonin. *Int. J. Mol. Sci.* 24:3022. 10.3390/ijms24033022 36769340 PMC9917989

[B115] Tocharus C. Puriboriboon Y. Junmanee T. Tocharus J. Ekthuwapranee K. Govitrapong P. (2014). Melatonin enhances adult rat hippocampal progenitor cell proliferation via ERK signaling pathway through melatonin receptor. *Neuroscience* 275 314–321. 10.1016/j.neuroscience.2014.06.026 24956284

[B116] Vallée A. Lecarpentier Y. (2016). Alzheimer disease: Crosstalk between the canonical Wnt/beta-catenin pathway and PPARs alpha and gamma. *Front. Neurosci.* 10:459. 10.3389/fnins.2016.00459 27807401 PMC5069291

[B117] Vázquez J. González B. Sempere V. Mas A. Torija M. J. Beltran G. (2017). Melatonin reduces oxidative stress damage induced by hydrogen peroxide in *Saccharomyces cerevisiae*. *Front. Microbiol.* 8:1066. 10.3389/fmicb.2017.01066 28663741 PMC5471302

[B118] Wang J. Gao S. Lenahan C. Gu Y. Wang X. Fang Y. (2022). Melatonin as an antioxidant agent in stroke: An updated review. *Aging Dis.* 13 1823–1844. 10.14336/AD.2022.0405 36465183 PMC9662272

[B119] Wang K. Ru J. Zhang H. Chen J. Lin X. Lin Z. (2020). Melatonin enhances the therapeutic effect of plasma exosomes against cerebral ischemia-induced pyroptosis through the TLR4/NF-κB pathway. *Front. Neurosci.* 14:848. 10.3389/fnins.2020.00848 33013286 PMC7461850

[B120] Wang Q. Wei S. Zhou H. Shen G. Gan X. Zhou S. (2019). Hyperglycemia exacerbates acetaminophen-induced acute liver injury by promoting liver-resident macrophage proinflammatory response via AMPK/PI3K/AKT-mediated oxidative stress. *Cell Death Discov.* 5:119. 10.1038/s41420-019-0198-y 31341645 PMC6642179

[B121] Wang Y. Luo J. Li S. Y. (2019). Nano-curcumin simultaneously protects the blood–brain barrier and reduces M1 microglial activation during cerebral ischemia–reperfusion injury. *ACS Appl. Mater. Interfaces* 11 3763–3770. 10.1021/acsami.8b20594 30618231

[B122] Wu C. X. Liu R. Gao M. Zhao G. Wu S. Wu C. F. (2013). Pinocembrin protects brain against ischemia/reperfusion injury by attenuating endoplasmic reticulum stress induced apoptosis. *Neurosci. Lett.* 546 57–62. 10.1016/j.neulet.2013.04.060 23669639

[B123] Wu M. Y. Yiang G. T. Liao W. T. Tsai A. P. Cheng Y. L. Cheng P. W. (2018). Current mechanistic concepts in ischemia and reperfusion injury. *Cell. Physiol. Biochem.* 46 1650–1667. 10.1159/000489241 29694958

[B124] Xie C. Ginet V. Sun Y. Koike M. Zhou K. Li T. (2016). Neuroprotection by selective neuronal deletion of Atg7 in neonatal brain injury. *Autophagy* 12 410–423. 10.1080/15548627.2015.1132134 26727396 PMC4835980

[B125] Xu X. Zhang L. Ye X. Hao Q. Zhang T. Cui G. (2018). Nrf2/ARE pathway inhibits ROS-induced NLRP3 inflammasome activation in BV2 cells after cerebral ischemia reperfusion. *Inflamm. Res.* 67 57–65. 10.1007/s00011-017-1095-6 28956063

[B126] Yang C. Hawkins K. E. Doré S. Candelario-Jalil E. (2019). Neuroinflammatory mechanisms of blood-brain barrier damage in ischemic stroke. *Am. J. Physiol. Cell Physiol.* 316 C135–C153. 10.1152/ajpcell.00136.2018 30379577 PMC6397344

[B127] Yang Y. Lu Y. Han F. Chang Y. Li X. Han Z. (2018). Saxagliptin regulates M1/M2 macrophage polarization via CaMKKβ/AMPK pathway to attenuate NAFLD. *Biochem. Biophys. Res. Commun.* 503 1618–1624. 10.1016/j.bbrc.2018.07.090 30060948

[B128] Yao M. Zhang L. Wang L. (2023). Astragaloside IV: A promising natural neuroprotective agent for neurological disorders. *Biomed. Pharmacother.* 159:114229. 10.1016/j.biopha.2023.114229 36652731

[B129] Yoon Y. M. Lee J. H. Song K. H. Noh H. Lee S. H. (2020). Melatonin-stimulated exosomes enhance the regenerative potential of chronic kidney disease-derived mesenchymal stem/stromal cells via cellular prion proteins. *J. Pineal Res.* 68:e12632. 10.1111/jpi.12632 31989677

[B130] Yu L. Li B. Zhang M. Jin Z. Duan W. Zhao G. (2016). Melatonin reduces PERK-eIF2α-ATF4-mediated endoplasmic reticulum stress during myocardial ischemia–reperfusion injury: Role of RISK and SAFE pathways interaction. *Apoptosis* 21 809–824. 10.1007/s10495-016-1246-1 27170343

[B131] Zha H. Fan Y. Yang L. Yin M. Miao W. He J. (2021). Autophagy protects against cerebral ischemic reperfusion injury by inhibiting neuroinflammation. *Am. J. Transl. Res.* 13:4726. PMC8205746 34150053

[B132] Zhang J. Fang X. Zhou Y. Deng X. Lu Y. Li J. (2015). The possible damaged mechanism and the preventive effect of monosialotetrahexosylganglioside in a rat model of cerebral ischemia-reperfusion injury. *J. Stroke Cerebrovasc. Dis.* 24 1471–1478. 10.1016/j.jstrokecerebrovasdis.2015.02.008 25934141

[B133] Zhang S. Sun W. C. Liang Z. D. Yin X. R. Ji Z. R. Chen X. H. (2020). LncRNA SNHG4 attenuates inflammatory responses by sponging miR-449c-5p and up-regulating STAT6 in microglial during cerebral ischemia-reperfusion injury. *Drug Design Dev. Ther.* 14 3683–3695. 10.2147/DDDT.S245445 32982175 PMC7494233

[B134] Zhang S. Wang P. Ren L. Hu C. Bi J. (2016). Protective effect of melatonin on soluble Aβ1–42-induced memory impairment, astrogliosis, and synaptic dysfunction via the Musashi1/Notch1/Hes1 signaling pathway in the rat hippocampus. *Alzheimers Res. Ther.* 8:40. 10.1186/s13195-016-0206-x 27630117 PMC5024520

[B135] Zhou X. Y. Luo Y. Zhu Y. M. Liu Z. H. Kent T. A. Rong J. G. (2017). Inhibition of autophagy blocks cathepsins-tBid-mitochondrial apoptotic signaling pathway via stabilization of lysosomal membrane in ischemic astrocytes. *Cell Death Dis.* 8:e2618. 10.1038/cddis.2017.34 28206988 PMC5386481

